# Art therapy masks reflect emotional changes in military personnel with PTSS

**DOI:** 10.1038/s41598-024-57128-5

**Published:** 2024-03-26

**Authors:** V. Estrada Gonzalez, V. Meletaki, M. Walker, J. Payano Sosa, A. Stamper, R. Srikanchana, J. L. King, K. Scott, E. R. Cardillo, C. Sours Rhodes, A. P. Christensen, K. M. Darda, C. I. Workman, A. Chatterjee

**Affiliations:** 1 Penn Center for Neuroaesthetics, Perelman School of Medicine, University of Pennsylvania, Philadelphia, USA; 2 National Intrepid Center of Excellence, Bethesda, USA; 3 National Endowment for the Arts, Washington, USA; 4 Henry M. Jackson Foundation for the Advancement of Military Medicine Inc., Bethesda, USA; 5 Department of Art Therapy, George Washington University, Washington, USA; 6 Department of Psychological and Brain Sciences, University of Delaware, Newark, USA; 7 Psychology and Human Development, Peabody College, Vanderbilt University, Nashville, USA; 8 School of Psychology, University of New South Wales, Sydney, Australia; 9 Advancement and Research in the Sciences and Arts (ARISA) Foundation, Pune, MH India

**Keywords:** Art therapy, Military personnel, Emotions, Post-traumatic stress symptoms, Psychology, Human behaviour

## Abstract

Among disabling post-traumatic stress symptoms (PTSS) are irritability, aggressive behavior, distressing memories and general impaired cognition and negative mood. Art therapy interventions, including mask-making, can potentially alleviate these symptoms. We tested the hypothesis that art conveys emotions and predicted that blinded viewers would be able to perceive changes in theoretically derived emotional profiles expressed in art made by military personnel with PTSS from the onset to the end of therapy. Five service members and veterans exhibiting PTSS were enrolled in an 8-session art therapy protocol, during which they artistically transformed papier-mâché masks at the beginning and end of the protocol. We found that blinded viewers without knowledge of the masks’ creation stage (onset or end of therapy) read initial masks as conveying more negative emotions (e.g., angry, upset, and challenged) and later masks as conveying more positive emotions (calm and pleasure). Based on the assessments from the blinded evaluators, we infer the emotional transition experienced by the participants was expressed in the masks. In an exploratory arm of the study, we also found that viewers were better able to empathize with the negative emotions experienced by participants with PTSS when asked to explicitly take their perspective.

## Introduction

Among the many beneficial uses of art, it can serve as a means of communication, a force for social cohesion, and a vehicle to express emotions. In this study, we focus on the emotionally expressive qualities of art as relevant to a population that experiences post-traumatic stress symptoms (PTSS).

PTSS affects a remarkable number of active-duty service members and veterans. According to the U.S. Department of Veteran Affairs, 29 out of 100 veterans are diagnosed with post-traumatic stress disorder (PTSD) at some point in their lives^1^. Finding effective interventions to address the emotional aftermath of traumatic experiences is crucial. King and Parada^2^ highlight art therapy as a promising approach to facilitate emotional regulation. Consistent with this view, Schouten et al.^3^ found that an 11-session art therapy program reduced intrusive thoughts and stress in people with PTSS. The program, which included drawing, painting, and collage-making, enhanced their optimism about the future. Campbell et al.^4^ demonstrated that participants who combined talk therapy with an art therapy program had a greater reduction in depression symptoms than those who participated in only talk therapy.

### PTSS and a non-verbal approach to treatment

PTSS involves re-experiencing trauma through intrusive thoughts and flashback memories. People suffering from this condition are hyper-vigilant and hyper-reactive to trauma-related stimuli, and experience difficulty concentrating, negative mood and sometimes have suicidal thoughts^5^. Tricyclic antidepressants, monoamine oxidase inhibitors, and serotonin reuptake inhibitors are commonly used to treat PTSS. While pharmacological approaches can improve PTSS, complete remission with drugs is unlikely^6,7^.

Cognitive behavioral therapy (CBT) is also a common treatment for PTSS. This verbal treatment focuses on identifying and changing negative patterns of thinking and behavior that can trigger PTSS. CBT is occasionally paired with pharmacological treatments to boost therapeutic efficacy^8^. Notably, the nonresponse rates of CBT for PTSS can be as high as 50%^9^. A comprehensive meta-analysis conducted by Bradley et al.^10^ revealed that 33% of patients continued to exhibit PTSS after CBT-focused psychotherapy. This persistence of symptoms increased to 44% when accounting for patients who prematurely discontinued the intervention. In addition to the specific challenges CBT addresses, individuals with PTSS may struggle with language impairments^11^. This symptom hinders psychotherapy because effective verbal communication is crucial to the intervention.

Exposure Therapy (ET), a specialized form of CBT, requires patients with PTSS to engage with and recount their traumatic experiences. Exposure therapy is predicated on the concept that engaging with traumatic memories through repeated focus helps to alter the emotional response to those memories, reducing the harm of recollection and thereby diminishing avoidant behavior^12^. A meta-analysis by Powers et al., encompassing 675 patients, found that ET is effective, with 86% of patients showing improvement in PTSS post-treatment^13^. However, the treatment has a high dropout rate, up to 32%^14^, and for veterans, the rate can reach 34%^15^, presumably because of the emotional challenges presented during the early stages of ET. Additionally, the efficacy of ET may not be sustained over time. A meta-analysis by Raghuraman et al. provided evidence of ET’s effectiveness in reducing PTS symptoms, but also indicated that treatment gains were not consistently sustained^16^. Furthermore, the data raise questions about ET’s comprehensive impact on PTSS. While depressive symptoms show some improvement, it’s uncertain if ET is equally effective across the full spectrum of PTSS.

In addition to pharmacological and CBT interventions, non-verbal approaches such as art therapy present an alternative strategy to reduce PTSS^17,18^. Art therapy allows people to express complex memories and emotions that might be challenging to articulate using language. In art therapy, people can externalize, reflect upon, gain insights into and ultimately transform their cognitive and emotional experiences^19–22^.

Of the various art therapy interventions, mask-making is commonly used with participants with PTSS. Patients are provided with a papier-mâché mask and invited to alter the mask with art materials^23^ (e.g., paint and three-dimensional elements such as clay and found objects). Mask-making offers a therapeutic avenue for people with PTSS, enabling them to express and confront distressing thoughts non-verbally^11^. For instance, a qualitative analysis of masks crafted by 307 active-duty service members by Walker and colleagues^22^ concluded that mask-making provides an opportunity to express aspects of identity, using symbols related to moral injuries, grief and loss, and a perceived disconnection with society.

Thus, mask-making may harness the power of creative expression to promote emotion regulation. When making masks, patients explore and express their emotions in a tangible and symbolic manner. Externalizing their inner thoughts and feelings offers a sense of control over their emotional experiences^11^.

If art making effectively channels emotional expression and art therapy facilitates emotional regulation in PTSS patients, then external viewers may witness the amelioration of PTSS symptoms by observing the transformation within the patients’ art. That is, as individuals with PTSS imprint their emotions onto the masks, those emotions might be discernible to viewers. In this study, we aimed to determine if masks created at the onset of the art therapy protocol express a different emotional profile compared to those created at its conclusion.

### The present study

**Figure 1:**
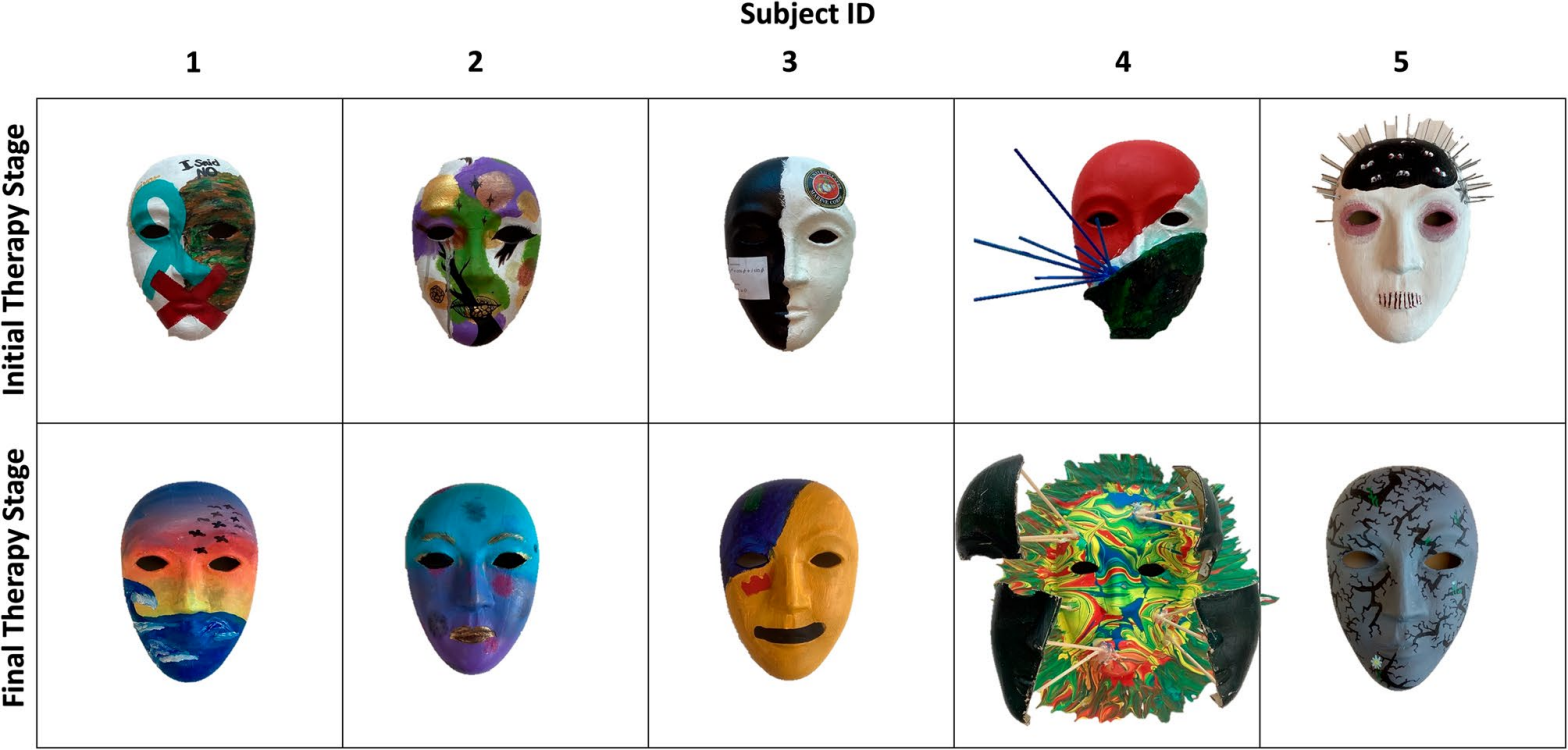
Masks painted by five military personnel participating in the art therapy intervention. Top masks were created during the initial therapy stage, whereas masks on the bottom were painted during the final therapy sessions. Masks are aligned per subject.

In this study, we presented 10 masks (Fig. 1) created by participants in an art therapy protocol to a separate group of individuals who were unaware of the treatment details. These independent observers were then asked to complete a questionnaire assessing the cognitive and emotional impacts elicited by the masks.

The masks were created as part of an 8-weeks art therapy research protocol facilitated by credentialed art therapists at The National Intrepid Center of Excellence (NICoE) at Walter Reed National Military Medical Center. The protocol parallels the interventions that active-duty service members receive within NICoE’s intensive and longitudinal outpatient programs^11,22,24–26^. The first two (initial) and last two art therapy sessions (final) comprise mask-making (Fig. 1). Service members and recently separated veterans who experienced PTSS (defined by ≥ 35 on the PCL-5) were recruited by NICoE to participate in this program.

These data are part of an ongoing clinical study that aims to recruit a larger sample of patients with PTSS^27^. Here, we focus on the emotional impact of the 10 masks (Fig. 1) felt by independent viewers, blinded to whether the masks were made at the initial or final therapy stage.

### Hypothesis and predictions

This study tested the hypothesis that artworks are a potent vehicle for emotional expression. We predicted that masks made by participants in this study would express emotional states and reflect a mitigation of negative emotional states in people with PTSS following treatment. To test this hypothesis, we invited raters to evaluate the impacts elicited by the masks^28^ created at the initial and final stages of the art therapy protocol. We predicted that observers unaware of when the masks were created or of the clinical details of participants would rate masks made in the final stage of therapy higher on positive emotional impacts compared to the masks created in the initial stage of the intervention. This approach is a strong test of the hypothesis since blinded viewers are not susceptible to biases that might be present when therapists and clinicians involved in care of patients interpret their art.

A core question in empirical aesthetics is how to assess the impacts of artwork on viewers beyond asking if people like art or find it interesting and beautiful. Our approach, as reported by Christensen et al.^28^, was to identify potential impacts of art by first querying 5 experts from psychology, philosophy, neuroscience, art history, and theology. From this expert group, 69 potential impact terms were derived, which were then subject to a crowd sourcing procedure in which non-expert participants generated associated terms. From the associations, using exploratory graph theory, a semantic network was derived that shows the interrelationship of the 69 terms. This network could be reduced to 11 dimensions: angry, calm, compassionate, challenged, edified, enraptured, enlightened, interested, inspired, pleasure, and upset. These dimensions could be further reduced into 4 categories of impacts- positive emotions (pleasure, calm, compassionate), negative emotions (angry, challenged, upset), motivation/immersion (interested, enraptured) and epistemic transformation (edified, enlightened, inspired). In addition to these dimensions, we also asked the raters how much they liked and how beautiful they found the masks to be.

We specifically predicted that masks created in the initial stage of the intervention would be read as expressing more negative emotional impacts, such as angry, upset, and challenged and that masks crafted during the final stage of the therapy would be rated more favorably in terms of positive impacts (pleasure, calm, compassionate). Such a shift would reflect the therapeutic progress of the participants. We were agnostic about whether changes in the motivation/immersion and the epistemic transformation impacts would be evident to blinded viewers.

A secondary aim of our study was to explore the role of perspective-taking in the perceived emotional expression of the masks. We drew from Vischer’s simulation theory for this purpose^29,30^. The theory postulates that an aesthetic experience is not a passive reception of sensory input. Instead, it involves an active, empathetic engagement with the artwork. This engagement is characterized by simulation, where the observer mentally recreates the emotional and imaginative state of the artist. We considered two perspectives based on this theory. The first one proposes that viewers automatically empathize with the mask creator’s emotions when looking at artworks. The second suggests that empathizing with the artist’s perspective is aided by external input, such as contextual cues or other prompts. To test these alternative hypotheses, we divided participants into two groups. One group was instructed to rate the emotional impact based on their own experience while viewing each mask. The other group was asked to rate the emotional impact based on their beliefs about what the artist might have felt while creating the artwork. If an empathetic reading of masks is automatic, then we would not detect any differences between the groups. However, if viewers frame their experience with an explicit consideration of the maker’s perspective, they would read the masks with a different emotional profile than when the masks were viewed without being guided to consider the maker.

## Results

### Intervention model

We compared the impact of masks created during the initial stage of art therapy with those created during the final stage by querying participants unaware of when the masks were made. To do so, we tested a model with two stages of therapy (i.e., ‘initial’ and ‘final’ therapy) as fixed effects. To control for effects of demographic variables, we added art experience (from our Art Experience Questionnaire), age, and years of education as fixed effects to the model. The Art Experience Questionnaire (AEQ) is designed to measure an individual’s experience and engagement with visual arts. It includes questions about educational background in studio art, art history, and art theory or aesthetics at the high school level or above. It also assesses the frequency of visits to art museums and galleries, time spent creating visual art, reading visual art-related publications, and looking at visual art on a weekly basis. All continuous variables were grand mean centered (e.g., AEQ scores).

$${\text{Intervention}}\;{\text{ Model}} < - {\text{ Rating }}\sim { 1 } + {\text{ therapy }}\;{\text{stage }} + {\text{ AEQ}} \; {\text{ score}} + {\text{ age }} + {\text{ education }} + { 1}|{\text{sid}}$$

Table 1 shows beta estimates, confidence intervals, and *p*-values for all predictors, including age and years of education. They provide information for liking and beauty ratings for the Intervention model.

**Table 1:** Estimates values, confidence intervals, and *p*-values for the intervention model for liking and beauty ratings.

Intervention model	Liking				Beauty			
Predictors	Estimates	CI	Statistic	*p*	Estimates	CI	Statistic	*p*
(Intercept)	3.5	3.38–3.61	59.44	** < 0.001**	3.31	3.21–3.42	59.96	** < 0.001**
Art Expertise	0.12	0.01–0.23	2.24	**0.025**	0.12	0.02–0.22	2.45	**0.014**
Age	0.12	0.01–0.22	2.2	**0.028**	0.01	− 0.08 to 0.11	0.23	0.818
Education	0.06	− 0.04 to 0.17	1.19	0.233	0.06	− 0.04 to 0.16	1.24	0.216
initial therapy stage	Reference				Reference			
final therapy stage	− 0.33	− 0.44 to − 0.23	− 6.5	** < 0.001**	− 0.7	− 0.80 to − 0.60	− 13.49	** < 0.001**
Marginal R^2^	0.036				0.079			

Significant *p* values are in bold.

The art therapy intervention affected ratings for liking and beauty and 10 impact dimensions: angry, upset challenged, calm, pleasure, enraptured, interested, and enlightened (liking: β =  − 0.33, *p* < 0.001; beauty: β =  − 0.7, *p* < 0.001; calm: β = − 0.17, *p* < 0.001; pleasure: β =  − 0.16, *p* = 0.009; angry: β =  − 0.47, *p* < 0.001; challenged: β = 0.2, *p* < 0.001; upset: β = 0.41, *p* < 0.001); interested: β = 0.09, *p* = 0.021; enraptured: β =  − 0.13, *p* = 0.029; enlightened: β = 0.25, *p* = 0.001.

**Figure 2:**
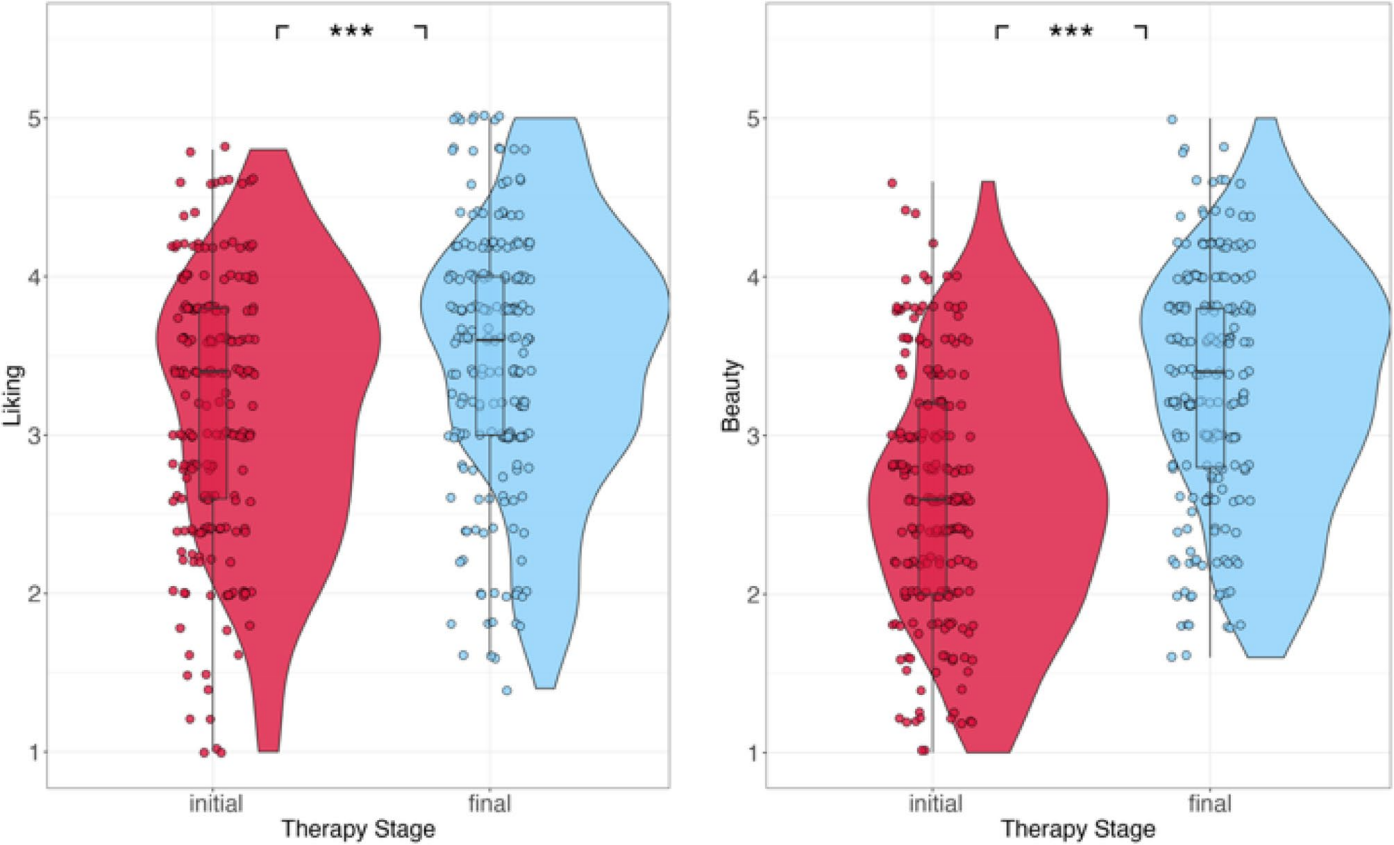
Liking (left) and beauty ratings (right) for initial and final therapy stages masks. *p < 0.05; **p < 0.01; ***p < 0.001.

Tukey post hoc t-tests revealed that participants liked and found more beautiful ‘final therapy stage’ masks more than the ‘initial therapy stage’ masks (Fig. 2; liking: estimate = 0.335, SE = 0.0515, *p* < 0.001; beauty: estimate = 0.697, SE = 0.0517, *p* < 0.001).

### Positive emotions

**Figure 3:**
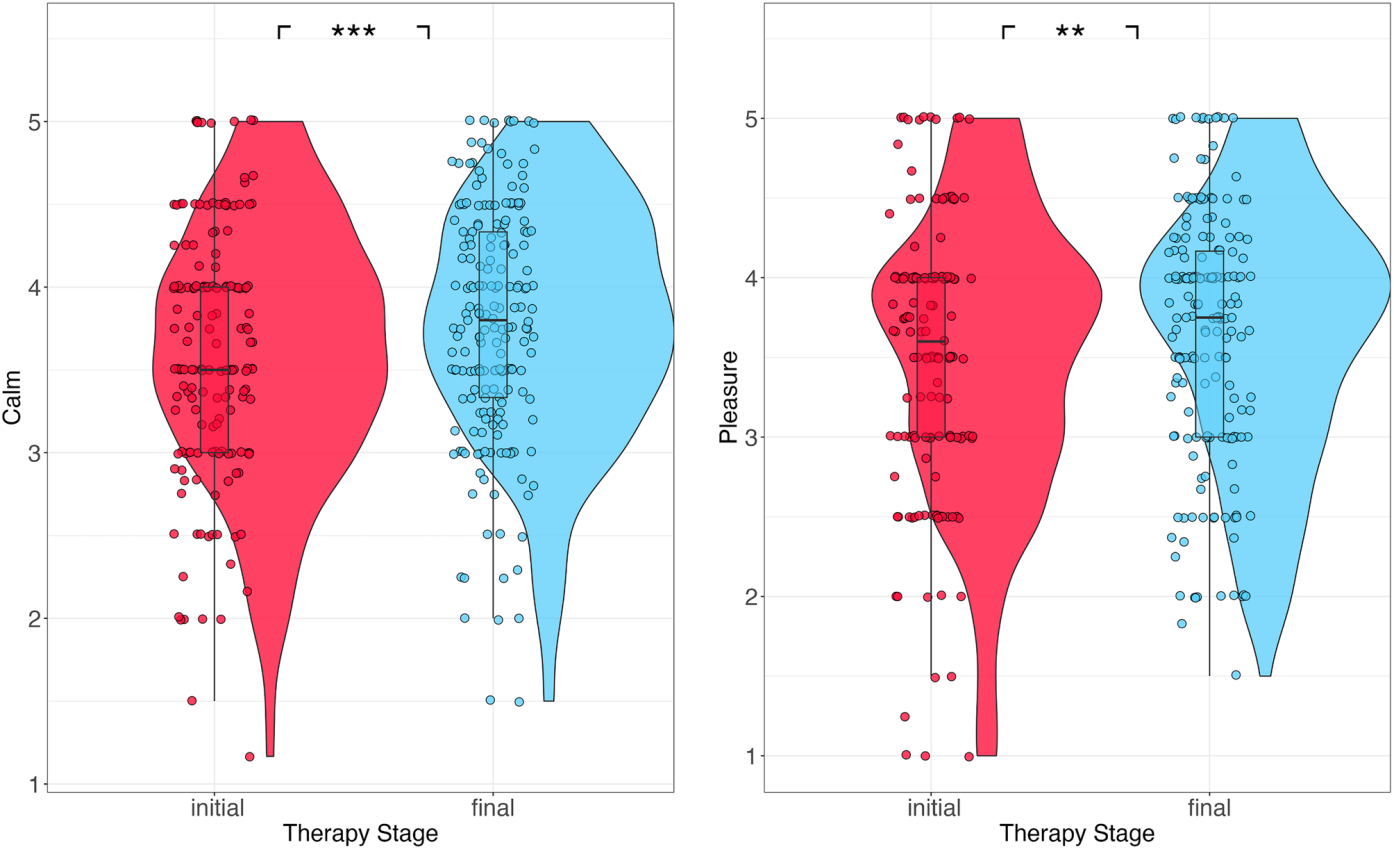
Calm (left) and pleasure ratings (right) for initial and final therapy stage masks. *p < 0.05; **p < 0.01; ***p < 0.001.

Tukey post hoc t-tests revealed that participants experienced ‘final therapy stage’ masks as inducing greater feelings of calmness and pleasure than initial masks (Fig. 3) calm: estimate = 0.174, SE = 0.0474, *p* = 0.003; pleasure: estimate = 0.156, SE = 0.0595, *p* = 0.009). Evaluations of compassionate were not significantly different between mask groups (compassionate: estimate = − 0.0721, SE = 0.13, *p* = 0.58).

### Negative emotions

**Figure 4:**
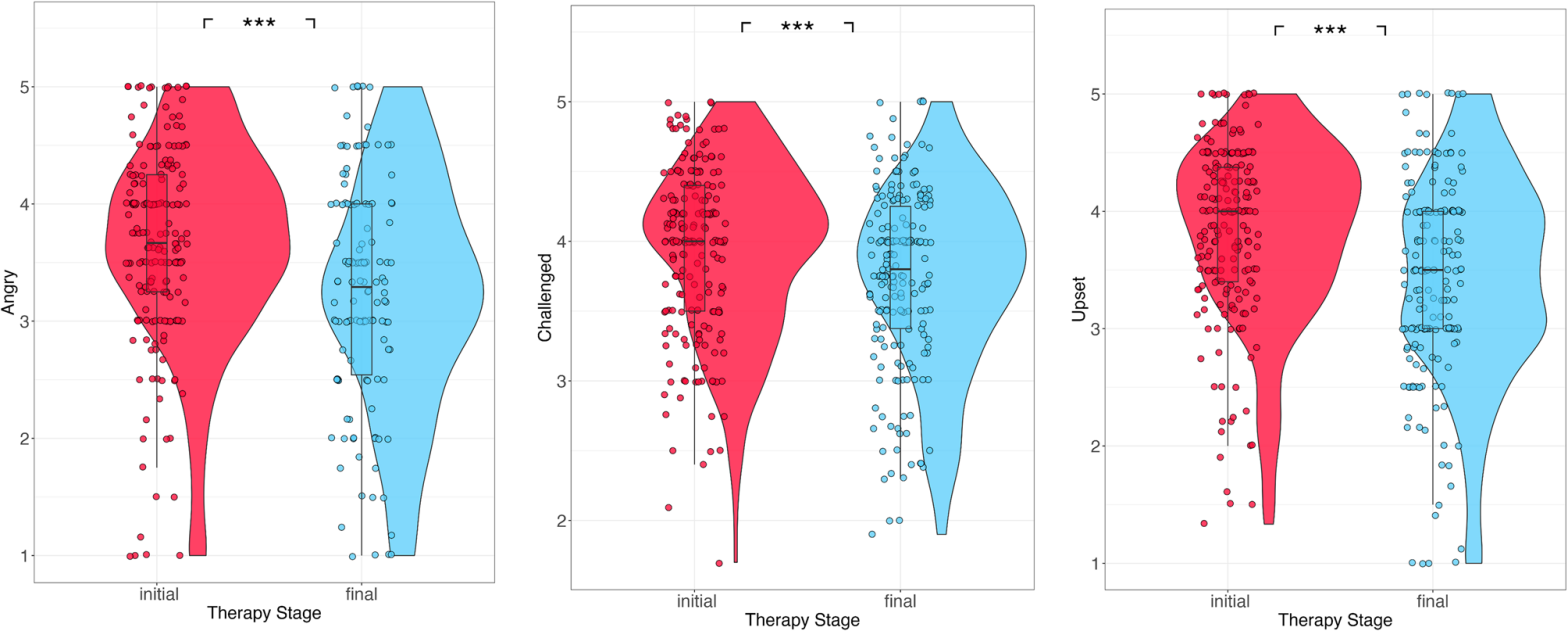
Angry (left), challenged (center), and upset (right) ratings for initial and final therapy stage masks. *p < 0.05; **p < 0.01; ***p < 0.001.

Participants also felt less angry, challenged, and upset in final than initial stage masks (Fig. 4; angry: estimate = − 0.466, SE = 0.067, *p* < 0.001; challenged: estimate = − 0.202, SE = 0.035, *p* = 0.001; upset: estimate = − 0.41, SE = 0.05, *p* < 0.001).

### Motivation/immersion

**Figure 5:**
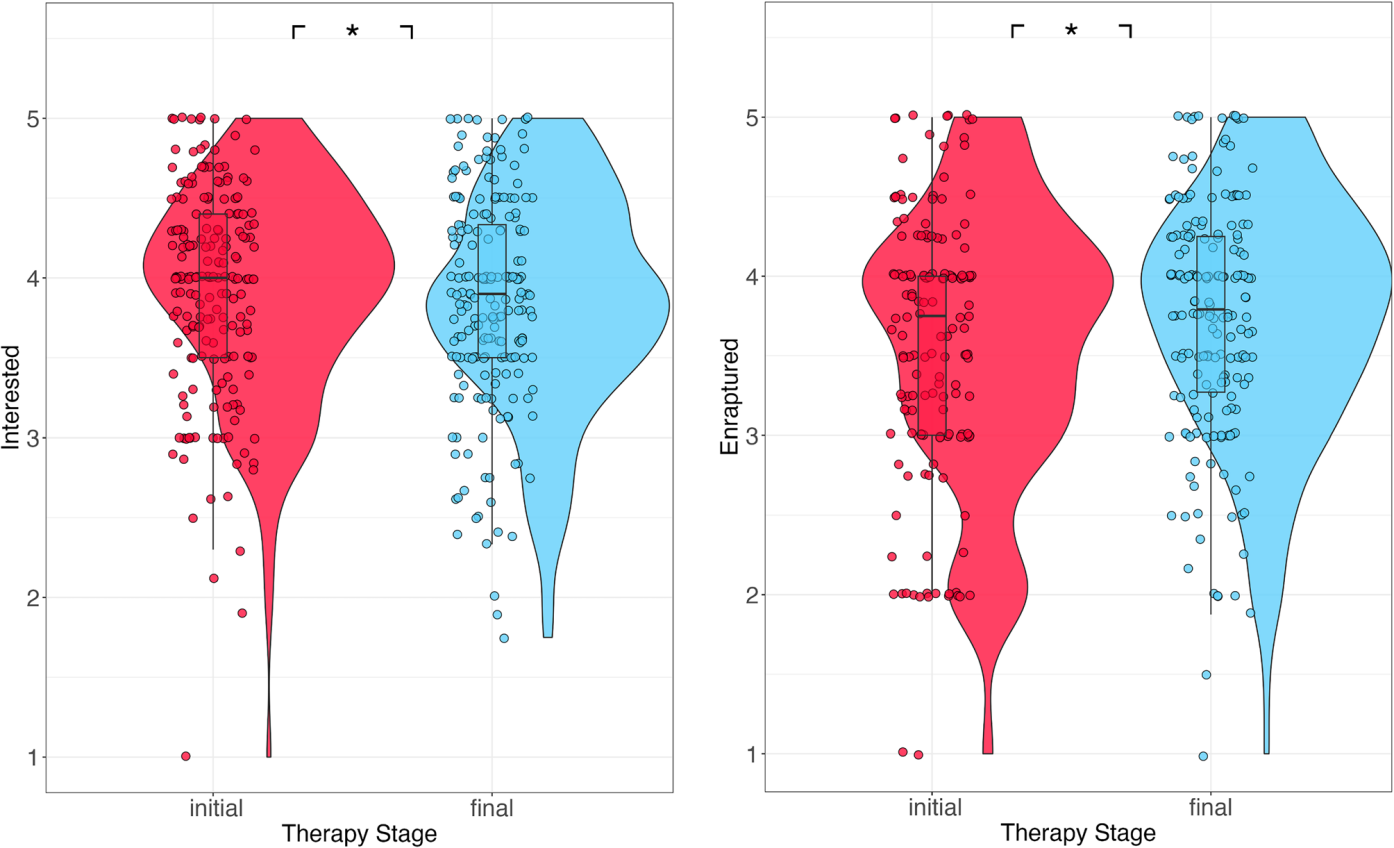
Interested (left) and Enraptured (right) ratings for initial and final therapy masks. *p < 0.05; **p < 0.01; ***p < 0.001.

Participants felt more interested in the initial stage than final stage masks. On the other hand, participants felt more enraptured towards the final stage than the initial one (Fig. 5; interested: estimate = − 0.0883, SE = 0.038, *p* = 0.021; enraptured: estimate = 0.127, SE = 0.0582, *p* = 0.0291).

### Epistemic transformation

Tukey-corrected post hoc tests showed no significant differences for edified and inspired ratings between the two therapy sessions (edified: estimate = − 0.0245, SE = 0.0613, *p* = 0.69; inspired: estimate = − 0.0263, SE = 0.0427, *p* = 0.537). Nevertheless, enlightened was significantly higher for the initial than for the final stage masks (enlightened: estimate = − 0.246, SE = 0.076, *p* = 0.001).

### Perspective-taking model

Our second aim was to investigate a strong or weaker version of an empathetic account of aesthetic engagement. We wished to find out if individuals’ subjective assessments of the masks were affected by their explicitly considering the emotional state of the artist when creating masks. One group of participants was asked about the impacts they experienced, while another group was inquired about what they believed the artist might have experienced while creating the mask.

For ratings of liking and beauty and each of the impacts (angry, calm, compassionate, challenged, edified, enraptured, enlightened, interested, inspired, pleasure, and upset), we ran a linear mixed effects model with perspective-taking as the fixed effect (rating of one’s perception or rating what the artist might have experienced), and by-subject random effects.

$${\text{Perspective - taking }} < - {\text{ Rating }}\sim { 1 } + {\text{condition }} + {\text{ age }} + {\text{ education }} + {\text{ AEQ}} \; {\text{ score }} + {1}|{\text{sid}}$$

Results showed that perspective-taking predicted liking, beauty, angry, challenged, interested, enlightened and inspired ratings (liking: β =  − 0.75, *p* < 0.001; beauty: β =  − 0.35, *p* < 0.001; angry: β =  − 0.3, *p* = 0.008; challenged: β =  − 0.17, *p* = 0.032; interested: β =  − 0.18, *p* = 0.031; enlightened: β =  − 0.22, *p* = 0.022; inspired: β =  − 0.32, *p* < 0.001).

Specifically, when participants were asked about the artists’ state of mind, they reported more liking (estimate = 0.75, SE = 0.0952, *p* < 0.001) and higher beauty ratings (estimate = 0.352, SE = 0.0977, *p* = 0.0004) compared to when they were asked about their personal impact while viewing them. Similarly, they inferred more anger (estimate = 0.299, SE = 0.112, *p* = 0.0085), challenge (estimate = 0.175, SE = 0.0814, *p* = 0.033), interest (estimate = 0.181, SE = 0.084, *p* = 0.0324) and inspiration (estimate = 0.325, SE = 0.0929, *p* = 0.0006) when considering the artist than how the masks impacted themselves.

No differences based on perspective-taking were found for calm (estimate = 0.157, SE = 0.0942, *p* = 0.096), compassionate (estimate = 0.0352, SE = 0.105, *p* = 0.7382), edified (estimate = 0.0747, SE = 0.112, *p* = 0.50), enraptured (estimate = 0.116, SE = 0.1, *p* = 0.26), enlightened (estimate = 0.18, SE = 0.103, *p* = 0.08), pleasure (estimate = 0.158, SE = 0.109, *p* = 0.1488) or upset (estimate = 0.152, SE = 0.102, *p* = 0.1372).

## Discussion

In this study, we tested the hypothesis that art serves as a vehicle for emotional expression in people with PTSS, whose distress can potentially be ameliorated by art therapy. The logic of our approach is based on the view that art offers an alternate means of emotional expression that can be difficult for people with PTSS to express verbally. Once externalized through art, their emotional expression may enhance well-being by providing a visual narrative of their internal experience. If this logic is accurate, one could see in the patients’ art the emotional change throughout the course of art therapy. Therefore, we predicted a viewer would be able to “read” changes in the impacts of the art produced from the beginning to the end of therapy.

Our study has two unique features. Firstly, we used aesthetic impact dimensions that offer a relatively granular assessment of the impact of the art beyond simply asking people if they liked the art or generally what emotions were being expressed. These impact dimensions were derived independently of any clinical intervention and generalized across different applications. Secondly, our viewers were blinded to whether the masks they assessed were made at the onset or at the end of the therapy sessions. While art therapists are trained to be sensitive to the symbolic and emotional meaning of the art patients produce, and their interpretations of the art are informed by their relationship with the person making the art, our viewers were agnostic to these factors and responded solely to the masks. As such, their responses are a more stringent assessment of the prediction that in military personnel with PTSS a change of emotional expression in art would be evident from the beginning to the end of therapy.

Masks crafted in the initial therapy stage were perceived to express more negative emotions than masks made in the final stage. The later masks were also thought to express more positive emotions than the early ones. These observations confirm our hypothesis that art making expresses emotions that are read by viewers and are consistent with the expected salutary effects of therapy on emotions in military personnel with PTSS. The observations from the masks predict that emotional regulation of participants with PTSS will improve with therapy. However, the findings reported here do not empirically validate this prediction with patients’ clinical data. Since our current sample includes only five patients, we plan to report detailed clinical data after enrolling a larger cohort, thereby adequately addressing our prediction.

To return to our specific findings, as anticipated, viewers experienced the initial masks to be more angry, upset, and challenging than the later masks. While people with PTSS often describe themselves as feeling numb, they were able to express negative emotions in their masks. Similarly, viewers found the final therapy stage masks expressed more calm and pleasure than the initial therapy stage masks. These observations are also consistent with the idea that the therapeutic intervention helped to balance their emotions.

While not predicted, viewers consistently rated the initial therapy stage masks as evoking more feelings of interest and enlightenment compared to the final therapy stage masks.

Our observations align with the ‘Distancing-Embracing’ model, which suggests that while art might depict or evoke negative emotions, viewers can still appreciate it from a safe psychological distance, allowing for both the experience of the emotion and the appreciation of the artistry^31^. The initial therapy stage masks, with their portrayal of intense negative emotions, might tap into this dynamic, offering viewers a raw yet distanced insight into human emotions^32,33^.

Finally, participants exhibited a clear preference for the masks created during the later stages of therapy, rating them as more beautiful and expressing a greater liking for them compared to those from the initial stages. This preference appears to be linked to the depth of engagement, as the enrapture ratings indicated that participants were more immersed in the experience of the final masks. These observations suggest that as therapy progressed, the masks not only served as a medium for emotional expression but also gained in artistic value, reflecting the transformative power of the therapeutic process.

The therapeutic goal of mask-making is to foster well-being in PTSS patients. Well-being encompasses not only the presence of positive emotions but also the capacity to understand and manage the entire spectrum of emotions—including negative emotions. Therefore, our findings do not imply that PTSS patients were solely experiencing positive emotions while creating the final stage masks. Instead, we report the dynamics of the thirteen emotional and cognitive impacts evaluated, illustrating a shift that may reflect a more complex emotional integration rather than a simple amelioration of negative emotions.

Our results contribute to the literature suggesting that mask-making can be a powerful intervention for individuals with PTSS^11,22^. The effectiveness of such interventions stems from the way art therapy empowers patients to articulate and process traumatic memories and emotions through non-verbal means. This claim is aligned with the literature^34^ suggesting that the process of creating and manipulating art provides individuals with a sense of control and agency over their emotional experiences, granting them this outlet for exploration and expression.

We also explored whether viewers’ perceptions of the masks aligned with assumptions about the emotional state of the artist making the masks. This experiment was aimed at understanding the role of perspective-taking, a cognitive process involving empathy with others’ emotional experiences, in the context of viewing art products created during art therapy (i.e., the masks). This exploration had a theoretical and a practical aim. The theoretical aim was to assess the validity of a strong version of the simulated empathy account. That is, do viewers automatically infer the emotions of the maker when they experience art? Or does explicitly taking the perspective of the artist change their reading of the art? The practical aim is that if we wish to advocate for treatment of people experiencing emotional distress, would others be more empathetic if they were guided explicitly to consider the state of the maker than if they engaged with the art without such guidance?

Our analysis revealed that people liked and found the masks more beautiful when considering the perspective of the artist than when not. We also found that perspective-taking affected ratings of the negative affective impacts of anger and challenge. When considering the maker, they also experienced more enlightenment, interest, and inspiration. None of the positive affective impacts (pleasure, calm, and compassion) were affected by considering the emotional state of the artist during mask creation.

Our observations generate a nuanced view of the role of empathy in art appreciation. Explicit perspective-taking may enhance general appreciation of the artwork with respect to liking and beauty. However, the art’s negative affective expression and immersive qualities are more susceptible to perspective-taking, possibly fostering a deeper understanding and empathy for the artist’s negative emotional experience. By contrast, perspective-taking did not influence impressions of positive affect such as calmness, compassion, and pleasure. This observation indicates that positive emotional responses may be less influenced by the perceived emotional state of the artist. These findings pave the way for future research to explore whether explicit perspective-taking affects negative emotions more than positive ones.

To sum up, blinded viewers perceived more negative emotions in the initial masks and more positive emotions in the final masks. This resonates with Schouten et al.’s^3^ work which showed that art therapy successfully reduced stress (negative emotional responses) and enhanced optimism (positive emotional responses). Therefore, our findings suggest that the emotional valence transition due to art therapy is embodied in the art expressions themselves. This finding underlines the value of integrating creative-based therapies within PTSS treatment strategies, particularly considering their lower dropout rates^38^, contrasting with the high drop-out rates of therapies such as ET^14^, and their unique capacity for facilitating the non-verbal processing of complex emotions^19–21^.

### Limits and further directions

This investigation opens several avenues for future research. One immediate direction involves expanding the study’s scope with a larger sample of artwork. Future studies could include a broader range of art samples as alternate means of emotional expression. By doing so, not only could one validate our findings regarding the expression of positive and negative emotions, but one could also investigate whether artworks from the initial stages of treatment continue to be perceived as more interesting and enlightening than those from later stages—as this was an unanticipated finding which needs to be replicated.

Importantly, we are not reporting the progress of PTSS patients receiving therapy, which limits our ability to directly correlate art therapy with clinical improvement. With a larger sample of participants, we could examine whether the masks rated positively at the therapy’s conclusion were made by patients with the greatest symptom improvement. Such an observation could further validate the therapeutic value of mask-making. While the Traumatic Brain Injury (TBI) levels of the military personnel were evaluated, we did not assess its impact on the therapeutic process or outcomes. Given the prevalence of TBI among veterans and its possible confounding effects on PTSS symptoms and treatment responses^39^, future research with a greater number of participants as we are planning to conduct could account for TBI when evaluating the efficacy of art therapy.

We advocate for a nuanced understanding of PTSS improvement, which involves emotional control rather than a simple shift from negative to positive emotions. Consequently, the improvement might not always manifest in the creation of art conveying positive emotions. Our taxonomy is not designed to measure the control of emotions. Further research could investigate whether improved emotional regulation in PTSS patients is also reflected in degree of changes in emotional expressivity in their artwork.

By expanding our inquiry into how mask making in art therapy reflects and influences the emotional journey of PTSS patients, we can deepen our understanding of the effects of art therapy and its manifestation in creative expression. This understanding could enhance clinical approaches to PTSS treatment.

### Clinical significance

This study contributes to the clinical understanding of therapeutic process in art therapy. Our findings suggest that art generated during therapy may act as a visual-emotional marker of patient state. Clinicians might find the analysis of these masks a valuable tool to map the therapeutic journey and evaluate treatment outcomes. At the same time, patients might also recognize evidence of their progress in the evolution of their own artworks and be motivated to continue with therapy.

Our findings support the usefulness of art therapy within the array of treatment options for PTSS. As one of relatively few non-verbal approaches available, art therapy holds particular significance for patients who struggle with verbal expression of their trauma. By highlighting positive outcomes, our study advocates for a broader, more versatile therapeutic approach that accommodates the needs of patients who may find treatments based on verbal communication challenging.

## Methods

### Art therapy intervention

Five service members and recently separated veterans, all with PCL-5 scores above 35 and without moderate to severe TBI, participated in the art therapy intervention. The protocol was developed at the National Intrepid Center of Excellence (NICoE) and consisted of the 8 sessions described in Table 2.

**Table 2:** Structure of the art therapy protocol conducted at the NICoE.

Session	Directive	Brief description
1	Initial therapy stage (mask-making)^24^	Participants were provided with a blank paper-mache mask template and instructed to alter the mask how they wish using paint and other art materials
2	Initial therapy stage (mask-making and writing prompt)^35,36^	Participants completed mask and wrote (15 min) using the prompt: “Create a title for your mask and describe what your mask conveys about you or your experiences including those related to TBI, PTSD, or other aspects of your military career.”
3	Bridge with Path drawing^37^	Participants chose a drawing medium and “Draw a bridge from someplace to someplace. The bridge connects to a path. Draw the path and write where the path leads you to.”
4	Box Project^24^	Participants were given an empty paper-mache box and asked to depict aspects of career and self on the outside of the box, and commemorate aspects of career, self, or lost friends inside the box using mixed media
5	Box project^24^	Participants finished the paper-mache box task
6	Pour painting	Participants chose symbolic acrylic paint colors, diluted and stacked the paint, spilled the paint onto a blank canvas, and maneuvered the canvas to create a marbling effect
7	Final therapy stage (mask-making)	Participants repeated Session 1 with a different mask
8	Final therapy stage (mask-making writing prompt)	Participants repeated Session 2 with the new mask

During the first two sessions (initial therapy stage) and the last two (final therapy stage), participants were instructed to “alter the mask how they wish using an array of art materials.” This study was approved by the Walter Reed National Military Medical Center Institutional Review Board and conducted in accordance with all Federal laws, regulations, and standards of practice as well as those of the Department of Defence and the Departments of Army/Navy/Air Force. In this study, we do not report any behavioral data about the veterans. The intervention is described with the purpose of understanding how the stimuli were created (i.e., face masks).

### Digital stimuli generation

The stimuli for this study comprised 10 digital images of the 5 masks painted during the initial therapy stage and the 5 masks painted during the final therapy stage (Fig. 1).

### Participants

240 English-speaking participants were recruited on Prolific (prolific.co) and provided informed consent before beginning. All procedures were approved by the University of Pennsylvania Institutional Review Board under protocol #806447.

At the end of the study, we asked participants whether the information they provided was good enough quality to be used (*Do you think the answers you’ve given here are honest and good enough quality that we should include them in our final analysis? You will still be paid no matter your response*). Participants who responded ‘No’ to the last question or those who did not finish the questionnaire (n = 37) were excluded from the experiment, leaving 203 participants in our analyses. The average age of participants was 38.2 years (SD = 12.19).

### Procedure

The blinded viewers were randomly assigned to one of two groups: Personal Impact (n = 100; 52 females) and Artist Impact (n = 103; 50 females). All participants were naïve to the primary goal of the study and did not know which masks were created at the beginning or the end of the art therapy protocol. While all participants viewed the same 10 painted masks, they received different instructions depending on their group. The personal impact group was instructed as follows:

*To review, your task is to rate how hand-painted masks make you think and feel. In total, you will see 10 different masks, each made by a different person. For each mask, ****you will be asked how it makes you think or feel. ****The image of the mask will be presented before each of these questions. In some cases, the meaning of two or more possible words describing your state might seem very similar. ****Please try to discern the ones that best fit your thoughts and feelings. You will also have the option to indicate that you think none of the words is relevant. ****You can look at the mask for as long or as little as you want. The survey will prompt you to move to the next slide when you are ready. Please click on the right arrow to proceed with the study.*

The Artist Impact group read the following:

*To review, your task is to rate what you believe the artist felt and thought while making a hand-painted mask. In total, you will see 10 different masks, each made by a different person****. For each mask, you will be asked questions about what you believe the artist thought and felt while making that mask.**** The image of the mask will be presented before each of these questions. In some cases, the meaning of two or more possible words describing the artist’s state might seem very similar. ****Please try to discern the ones that best fit your opinion of the artist’s state****. If you think none of the words are relevant, select “none apply.” You can look at the mask for as long or as little as you want. The survey will prompt you to move to the next slide when you are ready. Please click on the right arrow to proceed with the study.*

Masks were displayed in a random order for each participant. Participants could look at each mask for as long as they wanted. After clicking *next,* participants rated the emotional impacts of the mask along 11 dimensions: angry, calm, compassionate, challenged, edified, enraptured, enlightened, interested, inspired, pleasure, upset. Each impact dimension is associated with 3–8 associated emotion words (for example, angry: *frightened, offended, revolted, abrasive, subversive, enraged*). Dimensions and their associated terms were taken from the Aesthetic Impact Taxonomy developed by Christensen, Cardillo and Chatterjee (2023). For each impact dimension, participants were presented with the list of associated emotion words and given instructions that varied by group assignment:

Personal Impact: [*Select the two terms that best describe how the mask you just saw makes you think or feel. Select “none of these applies” if no words seem appropriate. This object makes me feel:*]

Artist impact: [*Select the two terms that describe the best what the artist felt and thought while making the last mask, in your opinion. Click “none of these applies” if no term is applicable. The artist felt:*]

After selecting the two most relevant emotion words, participants were instructed to rate the intensity of those emotions on a 5-point scale (1 = *only a little*; 5 = *great deal*). After the rating task, participants completed the Art Experience Questionnaire (AEQ) and a demographic questionnaire.

### Data analysis

Data pre-processing, statistical analyses, and data visualizations were performed using v.R4.2.2. Mixed effects models were conducted using the lme4 package (v.1.1.31). Post hoc tests were executed using the emmeans package (v.1.8.5). We used an α = 0.05 to make inferences and controlled for multiple comparisons using Tukey-HSD correction for post hoc tests.

For each of our dependent variables (average intensity ratings of emotion words within each impact dimension), we ran a linear mixed effects model. For the Intervention Model, the therapy stage (initial and final therapy stage masks) was added as the fixed effect and by-subject and by-item random effects. To control for the effects of demographic variables and art experience, we further added age, education, and AEQ score as fixed effects to the model. All continuous variables were centered to the mean by subtracting the mean from every value of the variable. The model was:

$${\text{Intervention }}\;{\text{Model }} < - {\text{ Rating }}\sim { 1 } + {\text{ therapy}}\;{\text{ stage }} + {\text{ AEQ}} \; {\text{ score}} + {\text{ age }} + {\text{ education }} + { 1}|{\text{sid}}$$

For the Perspective-taking model, the condition (artists’ impact and personal impact) was added as the fixed effect, alongside age, education, and total AEQ (art experience questionnaire) scores. By-subject was added as random effects.

$${\text{Perspective - taking }} < - {\text{ Rating }}\sim {\text{ 1 }} + {\text{condition }} + {\text{ age }} + {\text{ education }} + {\text{ AEQ}} \; {\text{ score }} + {\text{1}}|{\text{sid}}$$

## Data Availability

The raw dataset generated and analyzed during the current study is available in: https://github.com/vstradag/masks-data/blob/a18668bf68bc4c05b1b7b258f9deff9cf5a9bed4/rawdata.csv.
